# The bacterial effectors EspG and EspG2 induce a destructive calpain activity that is kept in check by the co-delivered Tir effector

**DOI:** 10.1111/j.1462-5822.2010.01469.x

**Published:** 2010-05-06

**Authors:** Paul Dean, Sabrina Mühlen, Sabine Quitard, Brendan Kenny

**Affiliations:** Institute for Cell and Molecular Biosciences, Medical School, University of NewcastleNewcastle-Upon-Tyne NE2 4HH, UK

## Abstract

Bacterial pathogens deliver multiple effector proteins into eukaryotic cells to subvert host cellular processes and an emerging theme is the cooperation between different effectors. Here, we reveal that a fine balance exists between effectors that are delivered by enteropathogenic *E. coli* (EPEC) which, if perturbed can have marked consequences on the outcome of the infection. We show that absence of the EPEC effector Tir confers onto the bacterium a potent ability to destroy polarized intestinal epithelia through extensive host cell detachment. This process was dependent on the EPEC effectors EspG and EspG2 through their activation of the host cysteine protease calpain. EspG and EspG2 are shown to activate calpain during EPEC infection, which increases significantly in the absence of Tir – leading to rapid host cell loss and necrosis. These findings reveal a new function for EspG and EspG2 and show that Tir, independent of its bacterial ligand Intimin, is essential for maintaining the integrity of the epithelium during EPEC infection by keeping the destructive activity of EspG and EspG2 in check.

## Introduction

Many of the world's most important diseases are caused by bacterial pathogens that deliver multiple effector proteins into eukaryotic host cells. Bacterial effector proteins are an evolutionary diverse family with a wide range of functions, enabling the bacterium to modulate many host cellular processes. Typically, individual effector proteins have a modular architecture with several functional domains or motifs, which confer multiple functions onto the effector. Emerging evidence suggests that effector proteins can cooperate with each other inside the host cell (Fu and Galan, 1999; McGhie *et al*., 2001; Kenny *et al*., 2002; Dean *et al*., 2006; Cain *et al*., 2008), giving the bacterial pathogen greater versatility in its ability to control host cellular events.

Enteropathogenic *E. coli* (EPEC) is a bacterial pathogen that causes severe watery diarrhoea, particularly in infants, and is responsible for a large proportion of infant deaths in the developing world (Chen and Frankel, 2005). Following ingestion, EPEC binds to the surface of the human small intestine where it delivers multiple effector proteins into small intestinal cells via a bacterial-encoded type III secretion system (T3SS). The best-characterized EPEC effector proteins are encoded in a genomic pathogenicity island called the locus of enterocyte effacement (LEE) which, in addition to the T3SS genes, carries at least six effector genes (*map*, *espF*, *espG*, *espZ*, *tir*, *espH*) and the *eae* gene that encodes the outer membrane protein Intimin (reviewed in Dean and Kenny, 2009). At least 14 effectors located outside the LEE region have been identified (Iguchi *et al*., 2009) although little is known about their function. Tir is the best-studied EPEC effector and is inserted into the host plasma membrane where it acts as a receptor for Intimin, mediating intimate bacterial attachment to the host cell (Kenny *et al*., 1997). Tir-Intimin interaction also induces actin-polymerization to form an actin-rich ‘pedestal’ beneath the bacterium. Several other Tir-dependent signalling events have been reported that all depend on Intimin (see Dean and Kenny, 2009). Other well-studied EPEC effectors are Map and EspF, which possess several overlapping functions as both disrupt tight junctions (TJs) (McNamara *et al*., 2001; Dean and Kenny, 2004), target mitochondria (Kenny and Jepson, 2000; Nougayrede and Donnenberg, 2004), efface intestinal microvilli (Dean *et al*., 2006) and inhibit the water transporter SGLT-1 (Dean *et al*., 2006) with EspF, but not Map, inhibiting phagocytosis (Quitard *et al*., 2006). In addition, Map has been shown to induce filopodia formation (Kenny *et al*., 2002) and promote cell invasion (Jepson *et al*., 2003) with the former event regulated by Tir through its interaction with Intimin. Much less is known about the functions of the other LEE effectorsalthough EspG and its non-LEE homologue EspG2 (also known as Orf3) have been shown to play a minor role in the disruption of epithelial barrier function (Tomson *et al*., 2005) and cause disruption of microtubules (Matsuzawa *et al*., 2004; Shaw *et al*., 2005a). EPEC has also been shown to cause an increase in levels of the general host cysteine protease calpain (Potter *et al*., 2003; Hardwidge *et al*., 2004), although the EPEC effectors that mediate calpain upregulation have not been determined. The calpains are a family of proteases that are found in many tissue types, including the ubiquitously expressed calpain I and II. Calpain cleaves a large number of host proteins, particularly proteins involved in host focal adhesions and cytoskeletal events and indeed calpain activity has been linked to cell migration and cell detachment (Glading *et al*., 2002).

It has long been established that EPEC can disrupt epithelial barrier function (Canil *et al*., 1993) – an essential feature of the intestinal epithelium that is maintained through apically located TJs between adjacent cells (Schneeberger and Lynch, 2004). TJs create an effective barrier to the movement of small molecules across the epithelium and this can be measured as *trans*-epithelial electrical resistance (TER). The disruption of TJs by EPEC causes a loss of TER and is dependent on the delivery of effector proteins into host cells (Guttman and Finlay, 2009). Previous studies have shown that several EPEC effectors contribute to epithelial barrier dysfunction including Map and EspF (McNamara *et al*., 2001; Dean and Kenny, 2004), with EspG and EspG2 playing a minor role (Tomson *et al*., 2005). While the outer membrane protein Intimin is essential for EPEC-mediated barrier disruption (Dean and Kenny, 2004), the contribution of its primary receptor Tir is unclear with previous studies reporting essential (Muza-Moons *et al*., 2003; Miyake *et al*., 2005) or non-essential (Dean and Kenny, 2004) roles. Recently, we deleted the *map*, *espF*, *eae* and *tir* genes in all combinations and found that the barrier-disrupting defects of all strains missing Map, EspF and/or Intimin were reversed by deleting the *tir* gene (P. Dean *et al*., unpublished). This supported previous findings that the *tir* mutant does indeed disrupt barrier function and also suggested that Tir possesses a novel role to prevent undefined effectors from causing barrier dysfunction.

Here, we show that disruption of epithelial barrier function by Tir-negative mutants involves an initial lag period, which provides an explanation of why previous reports have failed to observe the barrier-disrupting capacity of the *tir* mutant. Moreover, we show that Tir plays a critical role in maintaining the integrity of the epithelial monolayer as its absence promotes extensive detachment of host cells. This destructive activity was shown to be mediated by the redundant functions of two effectors – EspG and EspG2 – linked to the activity of the general host protease, calpain. EspG and EspG2 are shown to activate calpain during normal EPEC infection but their damaging effects on epithelial integrity are kept in-check by the Tir effector protein. This work highlights the delicate balance that has evolved between EPEC effectors within host cells and uncovers new functions for Tir and the EspG homologues.

## Results

### The *tir* mutant causes potent disruption of epithelial barrier function, independent of Intimin

**Fig. 1:**
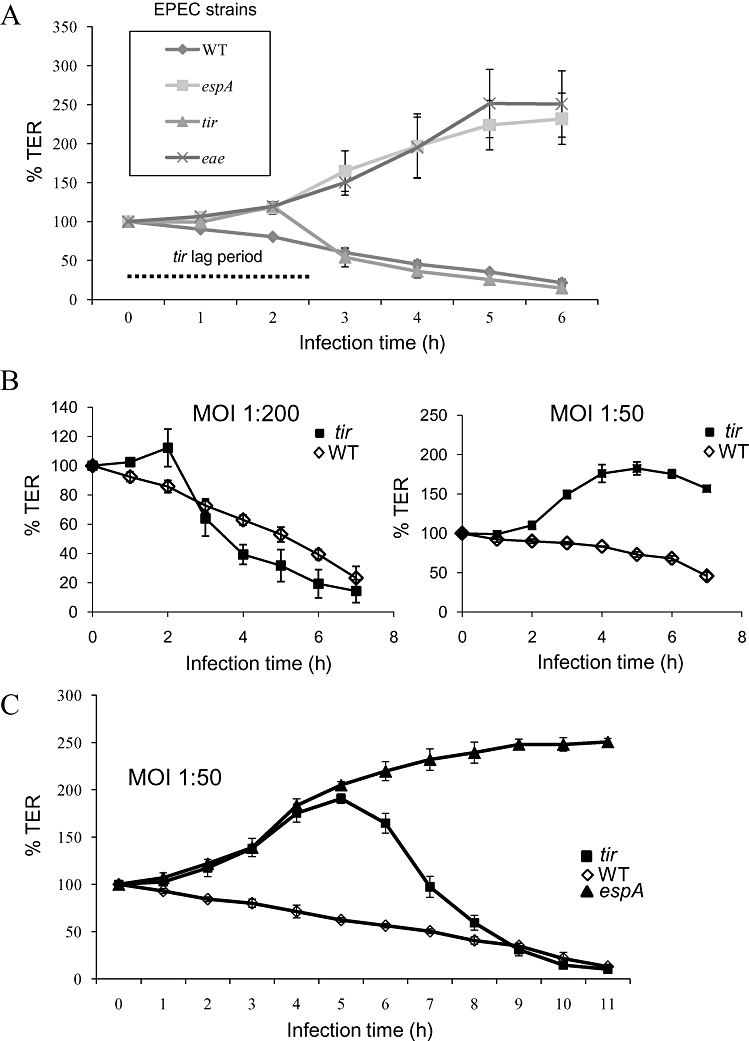
A lag phase persists in the disruption of barrier function by the *tir* mutant that is dictated by the multiplicity of infection (MOI). A. Polarized intestinal cells were infected with the indicated EPEC strains and the transepithelial resistance (TER) was followed. The dotted line indicates the lag period for the *tir* mutant where TER increases. The *eae* (Intimin) mutant cannot disrupt TER and was similar to that seen with the *espA* mutant (which cannot deliver effectors into the host cell). Points represent mean ± SEM, *n* = 3. B. Polarized intestinal cells were infected with wild-type (WT) EPEC or the *tir* mutant at the moi indicated and the transepithelial resistance (TER) was followed. The lag phase seen with the *tir* mutant at the moi of 1:200 (left) persisted to the end of the 7 h experiment at the moi of 1:50 (right). C. Increased infection times with the moi of 1:50 shows the *tir* mutant can cause a rapid TER decrease. The rate of TER decrease for the *tir* mutant following the lag period (35.11 h^−1^ ± 3.78 h^−1^) was significantly greater (*P* < 0.0001) than that of the WT (5.22 h^−1^ ± 1.52) while the *espA* mutant displayed no TER decrease. Data points show mean ± SEM, *n* = 3.

While our previous work revealed that *tir*–minus mutants disrupt epithelial barrier function to a level observed with wild-type (WT) EPEC, examination of the kinetics revealed a subtle difference. Thus, while EPEC infection of TC-7 polarized epithelial cells led to a gradual loss in transepithelial resistance (TER; a well-defined indicator of barrier disruption), a 1–2 h lag period consistently occurred with the *tir* mutant before it induced a rapid response (Fig. 1A). A similar result, whereby loss of barrier function was preceded by a lag period with the *tir* mutant, was also obtained with colonic T84 cells (not shown). As previously reported (Dean and Kenny, 2004), mutants missing a functional effector delivery system (*espA* strain) or the Intimin outer membrane protein (*eae* strain) are defective at causing barrier dysfunction and instead cause a progressive increase in TER values (Fig. 1A). The TER increase observed with these strains was due to increased pH of the growth medium during bacterial infection (not shown). Our studies routinely use a multiplicity of infection (moi) of 200 bacteria per host cell (Dean and Kenny, 2004; Dean *et al*., 2006), but much lower moi were used in studies reporting that the *tir* mutant does not decrease TER (Muza-Moons *et al*., 2003; Miyake *et al*., 2005). We examined whether the discrepancy in data was related to the differences in moi between the studies. A range of moi were tested (not shown) and an moi as high as 1:50 reproduced the findings of other studies (Fig. 1B), with the *tir* mutant, unlike the parental EPEC strain, failing to disrupt barrier function over the examined 6–7 h infection period (Muza-Moons *et al*., 2003; Miyake *et al*., 2005). By extending the infection time to 11 h, the *tir* mutant induced a rapid loss in barrier function after the lag, which occurred at a rate over 6 times faster (*P* < 0.0001) than the decrease with WT EPEC (Fig. 1C), while a TER decrease was not observed with the *espA* mutant over a similar 11 h infection period (Fig. 1C). Thus, these data clearly demonstrate that the *tir* mutant disrupts barrier function and shows that at lower bacterial doses, the lag period associated with the *tir* mutant persists for longer times, obscuring the strain's barrier-disrupting capacity.

### The absence of Tir causes destruction of the epithelium during infection

**Fig. 2:**
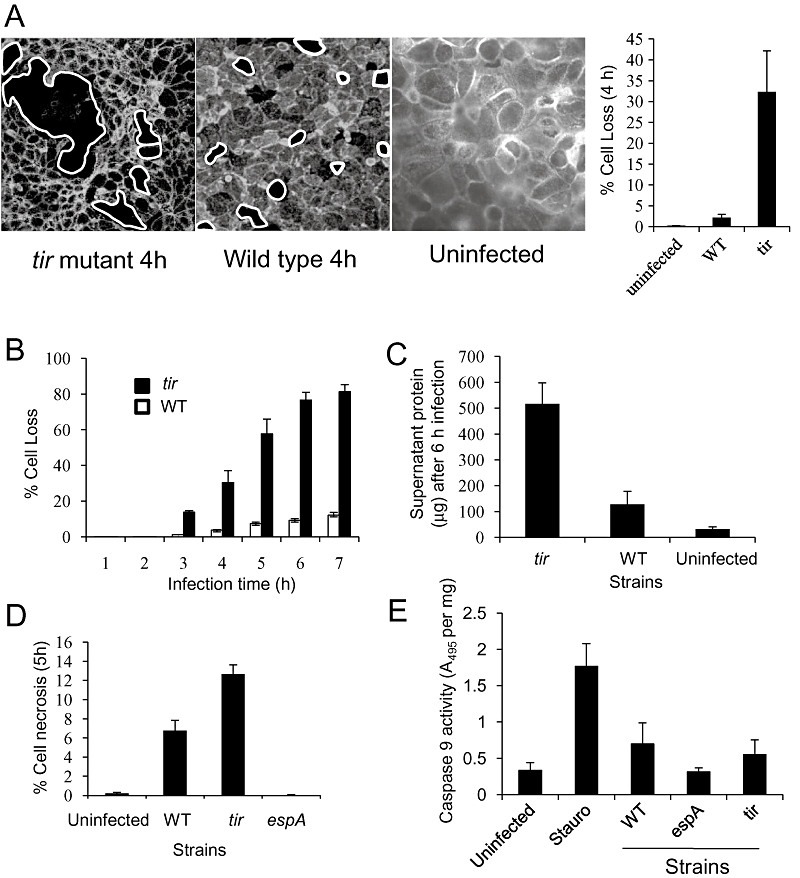
The absence of Tir causes a loss of epithelial monolayer integrity. A. Microscopy of polarized intestinal cells stained with phalloidin following a 4 h infection with the *tir* mutant or wild-type (WT) EPEC shows that *tir*-infected cells exhibit large cell-free voids in the monolayer, unlike that of WT. Percentage cell loss/unit area at 4 h post infection is shown in the right graph. B. Kinetics of cell loss during a 7 h infection with WT EPEC or the *tir* mutant for seven fields of view at ×63 per experiment. C. Cellular debris assessed by protein determination in the supernatant above the monolayer following a 6 h infection. D. Level of necrosis in monolayers infected with indicated strains determined by trypan blue staining (6 fields of view at ×40, *n* = 3). E. Levels of apoptosis in monolayers after 6 h infection with indicated EPEC strains assessed by caspase-9 cleavage activity per mg protein. Staurosporine (Stauro) was used as a positive control for apoptosis. For all graphs, data points represent mean ± SEM, *n* = 3.

Given the different kinetics of TER decrease between the *tir* mutant and WT EPEC, we postulated that a different mechanism of barrier dysfunction may be involved. Microscopy analysis of infected cells revealed large voids in *tir*-infected monolayers by 4 h post infection that was suggestive of cell detachment (Fig. 2A) while WT EPEC-infected monolayers remained relatively intact (Fig. 2A). Indeed, by 7 h post infection, less than 20% of the monolayer remained attached following *tir* mutant infections, unlike ∼90% coverage for monolayers infected with WT EPEC (Fig. 2B). Supporting these data, a corresponding (∼fivefold) increase of cellular debris (measured by protein determination) was evident in the supernatant of monolayers infected with the *tir* versus the WT EPEC strain (Fig. 2C, *P* < 0.001), suggesting that the epithelium was being rapidly destroyed during *tir* mutant infections. Examination of host cell death prior to this extensive cell loss (4 h post infection) revealed that WT EPEC infection was linked to low levels of necrosis (assessed by trypan blue) and apoptosis (assessed by caspase-9 cleavage). However, the *tir* mutant infection resulted in a ∼twofold significant increase in necrosis (Fig. 2D, *P* < 0.002) without impacting on the low level of apoptosis (Fig. 2E, *P* = 0.4). These data demonstrate that the rapid disruption of epithelial barrier function observed with the *tir* mutant is strongly correlated to detachment of host cells and linked to a necrotic response, suggesting that the Tir effector normally suppresses these events during EPEC infection.

### Pre-delivery of Tir is protective against the destructive activity of tir-deficient strains

**Fig. 3:**
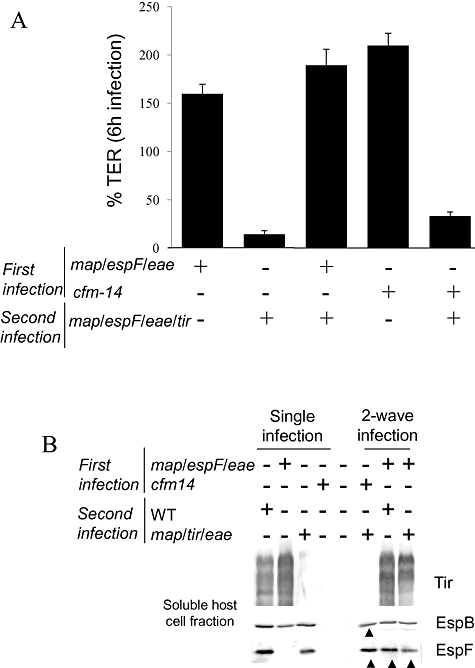
Pre-delivery of Tir into host cells protects the epithelium against the destructive activity of *tir*-minus mutants. A. Transepithelial resistance (TER) of polarized intestinal cells after 6 h single- or co-infection with indicated strains. For co-infections, monolayers were infected for 1 h with the triple *map/espF/eae* mutant (to deliver Tir into host cells) or *cfm-14* (a type three secretion mutant that cannot deliver Tir), which were then killed with antibiotics. Monolayers were then infected with the quadruple *map/espF/eae/tir* mutant for 6 h. Data points represent mean ± SEM, *n* = 3. B. Western blot of infected cell lysate from cells infected with first wave and second wave infections. Black arrow heads show that the first wave of infections does not affect the delivery of effectors by the strains used in the second wave of infections. A *map/tir/eae* triple mutant was used in place of the *map/espF/eae/tir* quadruple mutant for the secondary infections to demonstrate that delivery of EspF is unaffected and was similar to single- or co-infections with WT EPEC.

During EPEC infection, Map, EspF and Intimin (*eae*) collectively mediate barrier dysfunction (Dean and Kenny, 2004) and as such a *map/espF/eae* triple mutant is unable to disrupt TER (Fig. 3). However, we have found that a quadruple mutant (deleted for these three genes and *tir*) behaves like a *tir* mutant in that it causes rapid disruption of TER after an initial lag period that is associated with cell detachment (not shown; but see Fig. 3). This suggests that in the absence of Tir, disruption of TER does not require EPEC's prominent barrier-disrupting proteins Map, EspF or Intimin. Therefore, we utilized the *map/espF/eae* triple mutant to pre-deliver Tir into host cells to investigate whether Tir delivered by this strain would prevent the quadruple mutant from decreasing TER. Epithelial monolayers were infected for 1 h with the *map/espF/eae* triple mutant or a strain (*cfm-14*; TTSS^-^) unable to deliver Tir, after which the bacteria were killed with antibiotics. Subsequent infection by the quadruple mutant revealed that prior infection with the *map/espF/eae* strain prevented barrier dysfunction while *cfm-14* did not, suggesting that pre-delivery of Tir was essential.

As it has been reported that infection of non-polarized cells with effector-delivery competent strains inhibits the delivery of effectors by subsequent infections (Mills *et al*., 2008), we investigated whether our above findings were due to a defect in effector delivery by the quadruple mutant during the secondary infection. Detection of similar amounts of EspF and EspB within cells (Fig. 3B) pre-infected with the *map/espF/eae* followed by infection with either WT EPEC or a *map*/*eae*/*tir* mutant confirms that the second wave of infections are not impeded in their ability to deliver effector proteins. In addition, we found that a secondary infection with WT EPEC following pre-infection with the *map*/*eae*/*tir* triple mutant caused TER to decrease (not shown), further supporting the premise that effector delivery is not impeded in polarized cells. Taken together, the data show that two-wave infections work efficiently with polarized cells unlike that reported for non-polarized cells. This validates our interpretation of the above finding – that the rapid disruption of TER by the *tir* mutant that is linked to cell detachment and necrosis can be prevented by pre-delivery of Tir into host cells.

### Barrier dysfunction by the *tir* mutant occurs independently of actin rearrangements, unlike that of wild-type EPEC

**Fig. 4:**
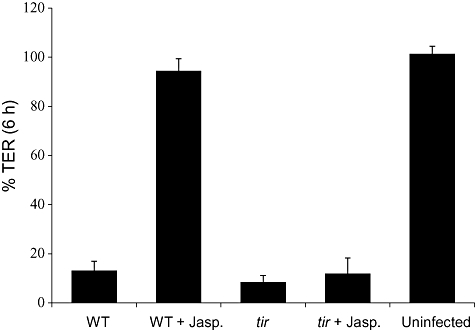
Loss of epithelial barrier function is dependent on actin rearrangements by wild-type (WT) EPEC but not the *tir* mutant. Transepithelial resistance (TER) of polarized intestinal cells after 6 h infection with WT EPEC or the *tir* mutant in the presence or absence of the actin stabilizing agent jasplakinolide. All data points are means ± SEM, *n* = 3. An moi of 1:200 was used for all experiments.

**Fig. 6:**
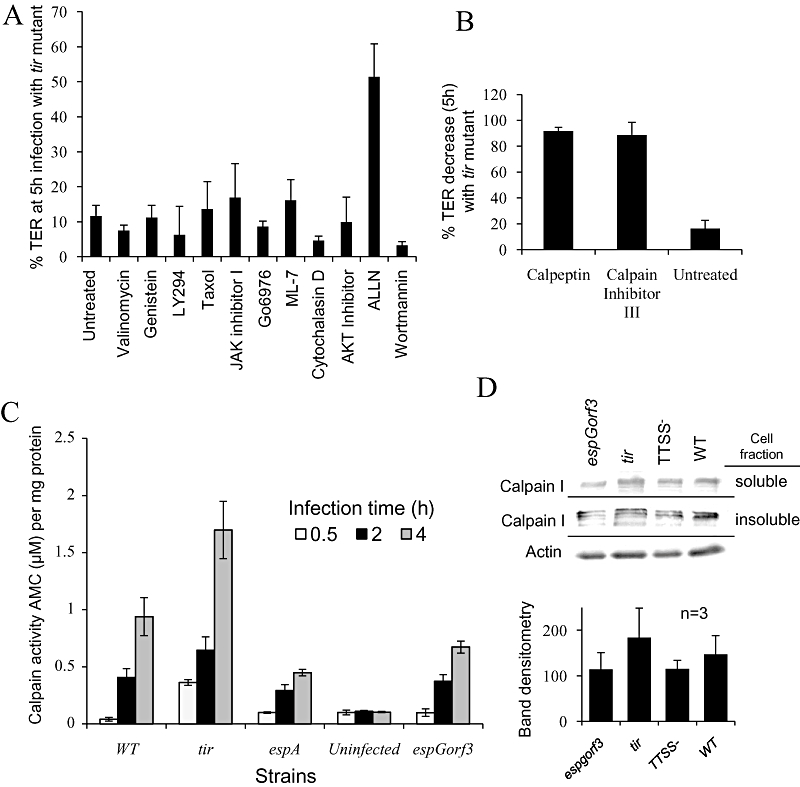
EspG and EspG2 over-activate calpain in the *tir* mutant. A. Inhibitor screening to identify the cellular pathway(s) involved in the potent *tir* mutant disruption of barrier function. Polarized intestinal cells were infected for 5 h with the *tir* mutant and transepithelial resistance (TER) was recorded. ALLN, a calpain inhibitor was the only inhibitor in this screen to significantly prevent the TER decrease. B. Two other calpain inhibitors calpeptin and calpain inhibitor III also prevented the TER decrease with the *tir* mutant. C. Calpain activity in cells infected with various EPEC strains was measured by the release of AMC from the calpain substrate Suc-LLVY-AMC per mg of cellular protein. All data points represent mean ± SEM, *n* = 3. D. Western blot of calpain I in host cells infected with the indicated strains. Calpain I bands were quantified by densitometry from three separate experiments. Bars show mean ± SEM.

We previously noted that disruption of TER by EPEC can be inhibited by pre-treating monolayers with the inhibitor jasplakinolide, which stabilizes actin filaments (not shown, but see Fig. 4). As the kinetics of TER decrease for WT EPEC and the *tir* mutant were different and were not dependent on Map, EspF and Intimin in the latter strain, we investigated whether the cellular mechanism was different for the two strains. Pre-treatment of cells with jasplakinolide prevented WT EPEC from decreasing TER (*P* < 0.0001) but had little, if any impact on the ability of the *tir* mutant to disrupt barrier function (*P* = 0.472; Fig. 4). Similar results were obtained with the actin-destabilizing agent cytochalasin D (not shown but see Fig. 6A). These data suggest that barrier disruption by WT EPEC is dependent on effector-driven actin rearrangements and also demonstrates that a different cellular process, independent of actin rearrangements, is responsible for TER decrease seen with the *tir* mutant.

### EspG and EspG2 mediate epithelial monolayer destruction in the absence of Tir

**Fig. 5:**
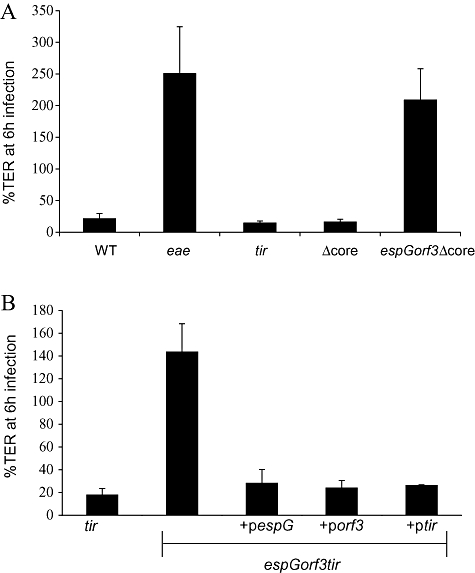
EspG and EspG2 are responsible for the loss of epithelial integrity caused by the absence of Tir. Transepithelial resistance (TER) of polarized intestinal cells infected for 6 h with indicated strains. A. Deletion of *espG* and *orf3* (encodes EspG2), but not other effector genes prevents the TER decrease associated with the *tir* mutant background. Δcore is unable to express or deliver the effectors Map, EspF, Tir, EspH, EspZ, NleA, NleH1, NleH2 and also absent for Intimin. *espG/orf3/*Δcore is absent for the effectors EspG and EspG2 in the Δcore mutant background. B. Complementation of the *espG/orf3/tir* triple mutant with plasmids carrying either *espG*, *orf3* (encodes EspG2) or *tir*. All infections were performed at moi of 1:200. Results show mean ± SEM, *n* = 3.

The ability of the *map/espF/eae/tir* quadruple mutant to cause detachment-linked TER decreases ruled out roles for Map, EspF and Intimin in the process. To investigate which effectors were involved, we examined the Δ*core* mutant that is missing *tir*, *map*, *espF* and *eae* (Intimin) genes and is also defective for the expression or secretion of additional effectors including EspH, EspZ, NleH, NleF and NleA (Ruchaud-Sparagano *et al*., 2007). The Δ*core* mutant mimicked the *tir* mutant in its ability to cause TER decrease (Fig. 5A, *P* = 0.7), suggesting the above described effectors were not involved. By contrast, studies with the *espG/orf3/*Δ*core* mutant (Ruchaud-Sparagano *et al*., 2007) implicated a critical role for the EspG homologues as this mutant was defective in causing a TER decrease (Fig. 5A). Subsequent involvement of EspG and EspG2 was supported by finding that an *espG/orf3/tir* triple mutant failed to decrease TER (Fig. 5B) unless complemented with plasmids encoding the *espG* or *orf3* genes (Fig. 5B). Interestingly, the inability of the *espG/orf3/tir* triple mutant (which carries intact *map*, *espF* and *eae* genes) to disrupt barrier function implicates a critical role for Tir in the well-defined Map/EspF/Intimin-dependent barrier disruptive pathway (Dean and Kenny, 2004). This premise was supported by restoring the strain's barrier-disrupting capacity by introducing a Tir-expressing plasmid (Fig. 5B). Thus, the data show that Tir (independent of Intimin) normally suppresses an EspG/EspG2 activity that leads to loss of TER by extensive cell detachment and also demonstrates that Tir (along with Intimin) plays a key, but previously cryptic, role in regulating the epithelial barrier disrupting functions of Map and EspF.

### EspG and EspG2's destructive activity is inhibited by calpain inhibitors

A reported function of the EspG homologues is linked to their destabilization of microtubules to release the RhoA-specific GEF (GEF/H1) that activates the RhoA-Rock signalling pathway to promote stress fibre formation (Matsuzawa *et al*., 2004). To investigate the role of this pathway in the TER decrease caused by the *tir* mutant, cells were pre-treated with the ROCK inhibitor Y27632. As this treatment had no impact on the process (not shown; but see below), we attempted to identify the signalling pathway(s) mediating the EspG destructive effect. An inhibitor screen was performed using a variety of broad spectrum or specific inhibitors (Table 1) based on signalling pathways implicated in EPEC subversive events. Consistent with the finding using the ROCK inhibitor, taxol – a microtubule stabilizing agent that prevents EspG-related RhoA activation (Matsuzawa *et al*., 2004; Tomson *et al*., 2005) – did not interfere with *tir* mutant TER decrease (Fig. 6A, *P* = 0.7). Thus, this suggests that the EspG-dependent activity linked to cell detachment is not related to the destabilization of microtubules or the triggering of the GEF/H1-RhoA-Rock signalling pathway. Strikingly, only one of the 12 interrogated inhibitors (ALLN; a non-specific cysteinyl protease inhibitor) prevented *tir* mutant-mediated TER decreases (Fig. 6A, *P* < 0.003). As a target of ALLN is ubiquitously expressed calpain – a Ca^2+^-dependent protease that regulates cytoskeletal anchorage complexes (Lebart and Benyamin, 2006) – we investigated this linkage using two calpain inhibitors (calpeptin and calpain inhibitor III). Both inhibitors were as effective as ALLN in preventing *tir* mutant-associated TER decreases (Fig. 6B), suggesting that calpain activity was involved in the TER decrease linked to monolayer destruction by the *tir* mutant. This also suggested that EspG and EspG2 may be activating calpain to cause host cell detachment in the absence of Tir. Similar results, in which EspG/Orf3 induce host cell detachment that is mediated through a calpain dependent pathway, were also obtained with HeLa cells (P. Dean *et al*., unpublished).

**Table 1:** Details of inhibitors used in the study.

Inhibitor	Working concentration for polarized TC-7 cells	Inhibitor site of action
Jasplakinolide	1 µM	Inhibits F-actin depolymerization
Cytochalasin D	5 µM	Inhibits F-actin polymerization
Wortmannin	1 µM	PI 3-kinase inhibitor
Valinomycin	1 µM	Disrupts mitochondrial membrane potential
AKT Inhibitor I	30 µM	AKT Inhibitor
Genistein	300 µM	Protein tyrosine kinase inhibitor
Go6976	5 µM	PKC inhibitor
ALLN	10 µM	Inhibits calpain I and II activity
ML-7	20 µM	Myosin light chain kinase inhibitor
Taxol	50 µM	Stabilizes microtubules
Staurosporine	1 µM	Broad spectrum protein kinase inhibitor, induces apoptosis
Calpeptin	50 µM	Inhibits calpain I and II activity
Calpain Inhibitor III	50 µM	Inhibits calpain I and II activity
LY294	25 µM	PI 3-kinase inhibitor
Y-27632	50 µM	ROCK inhibitor
JAK inhibitor I	16 µM	Inhibits Janus protein tyrosine kinases
Cyclohexamide	200 µg ml^−1^	Inhibits protein translation

### EspG and EspG2 over-activate host calpain activity in the absence of Tir

To explore the hypothesis that the EspG homologues promote calpain activation and that Tir may regulate this event, we examined cellular calpain activity. These studies used the synthetic calpain substrate Suc-LLVY-AMC at 4 h post infection (i.e. prior to extensive cell loss; Fig. 2). Figure 6C shows limited calpain activity in uninfected cells with the *espA* mutant causing a near-significant increase by 4 h post infection (*P* = 0.07; Fig. 1). Interestingly, while WT EPEC infection significantly increased calpain activity relative to *espA*-infected cells (*P* < 0.001), there was no significant difference between activity in *espG/orf3*- and *espA*-infected cells (*P* = 0.3). By contrast, cells infected with the *tir* mutant exhibited a significant (∼twofold) increase in calpain activity compared with WT EPEC (*P* < 0.001; Fig. 6C). This clearly shows that EPEC induce calpain activation in polarized epithelial cells in a T3SS-dependent manner through the effectors EspG and EspG2 and that this activation is modulated by Tir.

As a previous report has shown an increase in calpain in EPEC-infected cells (Hardwidge *et al*., 2004), we examined the levels of calpain in infected host cells to determine whether EspG/EspG2 induced a specific upregulation of calpain. Although the levels of the calpain I isoform were higher in the soluble and insoluble fractions of *tir*- and WT-infected cells as assessed by Western blot (Fig. 6D), their levels were not significantly different to the TTSS^-^ negative control (*P* = 0.2 and 0.31 respectively), unlike the significant difference shown in the calpain activity assay (Fig. 6C). Similar results were obtained for calpain II (ubiquitously expressed) and calpain V (found in intestinal tissue) (not shown), suggesting that upregulation of calpain levels in the *tir* mutant may not significantly contribute to the increased activation of calpain. Collectively, this study demonstrates that EspG/EspG2 promotes calpain activation during normal EPEC infection, which is kept under tight control by Tir to limit the cell detachment effects mediated by calpain.

## Discussion

While it is well established that bacterial effectors co-delivered into host cells can possess overlapping functions, an emerging property is their ability to cooperate with each other. Examples of this feature relate to effector-driven cytoskeletal rearrangements that are downregulated through the antagonistic action of co-delivered effectors (Fu and Galan, 1999; Kenny *et al*., 2002; Cain *et al*., 2008). However, these studies were performed on cell types that lack several key features of cells that are normally targeted by the bacteria *in vivo*. Here, we have used a disease-relevant small intestinal model to show that EPEC regulates the activity of the general host protease calpain through the cooperation of three of its effectors to ensure that extensive detachment of host cells is minimized during infection.

For over a decade it has been known that EPEC can disrupt epithelial barrier function by a mechanism dependent on the outer membrane protein Intimin (Canil *et al*., 1993). Further studies have determined that the disruptive process involves the activities of multiple effectors including prominent roles for Map and EspF and minor roles for the two EspG homologues (McNamara *et al*., 2001; Dean and Kenny, 2004; Tomson *et al*., 2005). Unexpectedly, a mutant missing the gene encoding the plasma membrane-located Tir effector, which is the main receptor for Intimin, was reported to be unable to disrupt barrier function (Muza-Moons *et al*., 2003; Miyake *et al*., 2005), while a separate study suggested otherwise (Dean and Kenny, 2004). In this study, we resolve this discrepancy by showing that although Tir has a critical role in EPEC's main barrier disrupting pathway (Map/EspF/Intimin-mediated), its contribution until now was cryptic because the absence of Tir leads to loss of barrier function by a completely separate mechanism. We show that previous studies which suggest that a *tir* mutant does not disrupt barrier function (Muza-Moons *et al*., 2003; Miyake *et al*., 2005) are due to modified infection protocols that resulted in low moi that unknowingly extended a lag period associated with the *tir* mutant, thereby obscuring the strain's barrier disrupting activity. These modified infection protocols used an moi as low as 1:3 (Miyake *et al*., 2005) or washed the epithelial cells after 1 h infection, thereby artificially reducing the moi (Muza-Moons *et al*., 2003). Our findings raise concerns on the interpretation of studies (Muza-Moons *et al*., 2003; Miyake *et al*., 2005; Tomson *et al*., 2005; Gill *et al*., 2007) with these modified infection protocols using mutants that exhibit lag periods in disrupting epithelial barrier function such as *tir* (this study) and *espG*/*orf3* mutants (Matsuzawa *et al*., 2005; Tomson *et al*., 2005; P. Dean *et al*., unpublished).

In this study, we show that the main barrier-disrupting proteins of EPEC are dependent on both Intimin and Tir to induce barrier dysfunction. This premise was derived from the finding that the *espG/orf3/tir* triple mutant is completely defective at causing a loss in TER much like the *eae* mutant, despite the presence of EspF and Map in these strains. This was further supported by finding that the ability of the *espG/orf3/tir* mutant to disrupt TER is restored when the strain is complemented with a Tir-encoding plasmid. Previously, this crucial role of Tir in barrier dysfunction was obscured in the single *tir* mutant (Dean and Kenny, 2004) because of the unknown destructive effects of EspG/G2 in this mutant background. Taken together, this study shows that EPEC-mediated barrier disruption requires Intimin (Canil *et al*., 1993; Dean and Kenny, 2004), Tir (this study) and the cooperative actions of Map and EspF (McNamara *et al*., 2001; Dean and Kenny, 2004). The finding that cytoskeletal inhibitors prevented WT EPEC but not the *tir* mutant from disrupting barrier function suggested that the aforementioned barrier-disrupting proteins function by altering the actin cytoskeleton. This fits well with the recently reported role of Map as a guanine nucleotide-exchange factor for Cdc42 – a regulator of cytoskeletal rearrangements (Huang *et al*., 2009), EspF as a direct and indirect activator of N-WASP to induce actin nucleation (Alto *et al*., 2007; Cheng *et al*., 2008; Peralta-Ramirez *et al*., 2008; Sallee *et al*., 2008) and Tir/Intimin in inducing actin rearrangements (Kenny *et al*., 1997). While studies using pharmacological agents have also implicated roles for host kinases in barrier dysfunction (Guttman and Finlay, 2009), it is likely that these drugs act by preventing or counter-acting cytoskeletal rearrangements triggered by these effectors. Recently, another EPEC effector NleA was reported to disrupt epithelial barrier function (Thanabalasuriar *et al*., 2010) and the work presented here suggests that NleA, like Map and EspF, cannot function without Intimin or Tir being present as shown by the complete defectiveness of the *eae* (Intimin) or *espG/orf3/tir* mutants.

Tir's role as a central regulatory effector must be given serious consideration in light of past reports and the data presented here. Tir has been shown to downregulate Map-induced filopodia formation (Kenny *et al*., 2002), modulate EspF's ability to efface microvilli (P. Dean *et al*., unpublished), is implicated in NleA-mediated disruption of barrier function (as described) and is shown to suppress EspG/G2's ability to activate calpain. Thus, in addition to its crucial role in intimate attachment (Marches *et al*., 2000), Tir appears to play an additionally important role in regulating the functions of multiple effectors involved in several patho-physiological events.

The *tir* mutant disrupted barrier function up to ∼6 times faster than the parental WT strain, suggesting that it employed an alternative cellular mechanism. Although all work in this study was performed with the Caco-2 derived TC-7 cells, we found a similar result with colonic T84 cells (not shown). Morphological and inhibitor data further supported the idea that the *tir*-negative mutant disrupts barrier function by a distinct pathway. Thus, whereas EPEC-mediated TER decreases are linked with TJ disassembly (Simonovic *et al*., 2000; Dean and Kenny, 2004; Guttman and Finlay, 2009), the *tir* mutant was linked with extensive cell detachment with up to ∼80% of the monolayer destroyed, compared with just ∼10% for WT EPEC. In addition, we found that the TJ complexes during *tir* mutant infection were intact in the host cells that remaining attached (not shown), unlike that with WT EPEC (Dean and Kenny, 2004). Thus, cell detachment provided an explanation for the rapid loss of TER associated with the *tir* mutant as monolayer confluency is directly linked with TER. The cellular mechanism that was responsible for the destructive phenotype associated with the *tir* mutant was dependent on the activation of host calpains as the TER decrease caused by the *tir* mutant was (i) prevented by inhibitors whose primary target are the Ca^2+^-dependent calpains and (ii) calpain activity was significantly higher in the *tir* mutant infected cells. The finding that the cell-detaching effects of the *tir* mutant were dependent on EspG and EspG2 and that it correlated with the increase in calpain activity, which was also dependent on these two effectors, strongly supports the contention that Tir acts to suppress EspG/EspG2-induced calpain activity. Interestingly, the Tir-EspG/EspG2 calpain pathway was not exclusive to polarized intestinal cells as we found a similar finding with HeLa cells (not shown). Recent structural data suggest that EspG family members might act as a scaffold for a papain-like cysteine protease with the N-terminus noted to be topologically similar to cysteine protease inhibitors (Davis *et al*., 2008). This provides a mechanism whereby EspG may recruit activated calpain but with specific calpain activity regulated through EspG's N-terminal domain, dependent on an unknown function of Tir.

Previously, EPEC was reported to cause a threefold to fivefold increase in the levels of calpain in intestinal cells (Hardwidge *et al*., 2004). We were unable to detect a similar increase in protein levels for three common calpain isoforms, although we did observe subtle differences between the levels of calpain in cells infected with WT EPEC, *espG/orf3* and the *tir* mutant. However, our finding that pre-treating host cells with cyclohexamide (which prevents protein translation) did not compromise *tir* mutant barrier disruption (not shown) suggests that the upregulation of calpain levels is not essential for the *tir* mutant to activate calpain. Further studies are underway to elucidate the mechanism by which Tir and EspG modulate host calpain activity and the contribution of the different calpain isoforms to the EPEC disease process.

Integrating the inhibitor, mutant and calpain activity data with published work offers a model whereby EPEC delivers the multi-functional EspG effectors to subvert various cellular processes including microtubule destabilization (Matsuzawa *et al*., 2005; Tomson *et al*., 2005), stress fibre formation (Matsuzawa *et al*., 2004), increasing paracellular permeability (Matsuzawa *et al*., 2005; Tomson *et al*., 2005), co-transporter inhibition (Gill *et al*., 2007) and inducing calpain activation (this study). We speculate that EPEC delivers sufficient levels of the EspG/EspG2 to modulate these cellular processes with a need for Tir to control or dampen the induction of calpain activity to prevent cell detachment. Calpain cleaves a number of host proteins, particularly those found in adhesion complexes and their cleavage results in disassembly of focal adhesions and cell rounding (Glading *et al*., 2002; Franco and Huttenlocher, 2005), thus the linkage between calpain and cell detachment is well established. Prevention of host cell detachment and limiting cell turnover is an important strategy of bacterial pathogens as host intestinal enterocytes are continually being replaced, preventing many harmful invaders from becoming established. Bacterial effector proteins have been identified that inhibit host cell turnover, including Cif of A/E pathogens, which blocks cell division (Marches *et al*., 2003), or the recently reported *Shigella* effector OspG, which prevents cell detachment by reinforcing focal adhesions in polarized epithelial cells (Kim *et al*., 2005). Our study suggests that the EPEC Tir can be included into the family of effectors that inhibit cell detachment.

What is the role of calpain activation during WT EPEC infection? Although EPEC causes detachment of non-polarized cells in a T3SS-dependent manner (Shifrin *et al*., 2002), this is not a prominent feature associated with EPEC-infected polarized epithelia, suggesting that the small increases in calpain activity induced by EspG/EspG2 in WT EPEC have other roles. It has been suggested that calpain plays a role in microvilli effacement by EPEC (Potter *et al*., 2003), but we and others find no role for EspG or EspG2 in effacement (P. Dean *et al*., unpublished; Shaw *et al*., 2005b). Equally, calpain has been implicated as a pro- and anti-apoptotic protein, but the low level of apoptotic cells induced by EPEC was similar for both the WT and the *tir* mutant. A more likely role relates to the cleavage of tight junctional proteins such as occludin (Zhu *et al*., 2006; Chun and Prince, 2009), which may explain EspG's reported role in selectively opening TJs to small molecules (Matsuzawa *et al*., 2005). However, the finding that Map and EspF cause a loss of the tight junctional protein occludin in infected cells (Dean and Kenny, 2004) and that an *espGorf3* double mutant causes significant tight junctional damage (Matsuzawa *et al*., 2005; Tomson *et al*., 2005) implies that EPEC interaction with TJs is a complex process with overlapping effector functions. Whatever the role, it is clear that the cleavage of host proteins by calpain is not very promiscuous during EPEC infection but is under tight control as cell detachment is not a common event, suggesting that the main target of calpain, the adhesion complexes, remains relatively intact during infection.

There is a growing consensus that interplay between bacterial effector proteins is highly complex and well-regulated process. While there are only a few examples of regulatory mechanisms it is clear that the functions of effectors do not proceed without restriction but are tight controlled by the bacterium. This makes sense as most effectors are multi-functional and have subtle effects on the host cell, which if left unchecked may become too deleterious, loosening the pathogen's grip on the host cell. Future studies will determine the unanswered questions that remain including the nature of Tir suppressive function on EspG/G2 activity, the mechanism of calpain induction by EspG/G2 and the role that calpain activity plays during EPEC infection.

## Experimental procedures

### Bacterial strains and plasmids

Enteropathogenic *E. coli* strains used in this study were WT EPEC (E2348/69 O127:H6) (Levine *et al*., 1985), *tir* (Kenny *et al*., 1997), *espA* (Kenny *et al*., 1996), *espG/orf3* (Elliott *et al*., 2001), *cfm-14* (Donnenberg *et al*., 1990), the *eae* (Intimin) mutant CVD206 (Donnenberg and Kaper, 1991), the quadruple mutant *map/espF/eae/tir* (Quitard *et al*., 2006) and the *espG/orf3/*Δ*core* mutant (Ruchaud-Sparagano *et al*., 2007). The Δ*core* mutant was made as per the *espG/orf3/*Δ*core* mutant (Ruchaud-Sparagano *et al*., 2007) using EPEC rather than the *espG*/*orf3* double mutant as a recipient for allelic exchange. The *map/espF/eae* triple mutant was generated via allelic exchange using the suicide vector pCVD442 (Donnenberg and Kaper, 1991) as described previously (Kenny *et al*., 1997). Briefly, the *map* mutant was used as a recipient for pCVD442-Δ*espF* to generate the *map/espF* double mutant, which was used as a recipient for pCVD442-Δ*eae* generating the *map/espF/eae* triple mutant. The triple mutant *espG/orf3/tir* was made by allelic exchange using the *espG/orf3* mutant as a recipient for the suicide vector pCVD442-Δ*tir*. Disruption of targeted gene was verified by PCR with Western blot analysis confirming that the strain had a functional T3SS and thus could deliver EspB into host cells. The *espG-* (Matsuzawa *et al*., 2005) and *tir*-encoding (Kenny, 2001) plasmids were selected with carbenicilin (100 µg ml^−1^ final concentration) or chloramphenicol (25 µg ml^−1^ final concentration) respectively.

### Cell culture and infection assays

Bacterial culture and infection assays were as described previously (Dean and Kenny, 2004). The TC-7 cell line (Chantret *et al*., 1994), a subclone of the heterogeneous intestinal cell line Caco-2, was used for all assays in this study. TC-7 cells provide a homogeneous population of enterocytes with a regular brush border and rapid doubling time (Chantret *et al*., 1994). This well-characterized cell line was used as it mimicked host–pathogen events better than the parent Caco-2 cell line with less inherent variability. TC-7 cells were used 12–15 days post confluence and cultured as previously described for Caco-2 cells (Dean and Kenny, 2004) in transwells (Corning) on membrane filters with 0.4 µM sized pore. Infection assays were performed as previously reported for Caco-2 (Dean and Kenny, 2004). Briefly, the OD_600_ of overnight bacterial cultures was measured and suspensions were diluted in Dulbecco's modified Eagle's medium (DMEM) and grown in this medium (at 37°C in 5% CO_2_) for 3 h. The OD_600_ of the suspensions was measured and diluted accordingly to the indicated moi (typically 1:200 for most experiments) and added to the apical surface of TC-7 monolayers on membrane filters. TER was measured at selected time points as previously described (Dean and Kenny, 2004) with the TER values for the TC-7 cell line consistently between 200 and 300 Ω cm^−2^. The term TER corresponds to the electrical resistance of the epithelium plus that of the membrane filter and solutions with TER fluctuation not providing mechanistic insight on the nature of any change.

### Western blot

Infected cells were washed twice in PBS and lysed in 1% (v/v) Triton-X 100 for 5 min prior to centrifugation at 12 000 *g*. The ‘soluble’ supernatant containing the cytoplasmic and membrane fractions was removed and the ‘insoluble’ cell pellet was washed in PBS. Both fractions were subject to SDS-PAGE and Western blot as described (Dean and Kenny, 2004). Antibodies used were calpain-µ (Sigma), calpain-V (Biovision), calpain-I (Chemicon) and actin (Sigma). To quantify the intensity of the bands, densitometrical analysis from at three separate blots was performed using the software program Image J.

### Two-wave infections

TC-7 cells (12–15 days post confluence) were left uninfected or infected 1 h with the *map/espF/eae* or *cfm-14* mutants (moi was 1:200). After infection the cells were washed three times in DMEM and the bacteria killed (gentamycin 100 µg ml^−1^ and penicillin/streptomycin 100 units ml^−1^ final concentration in DMEM for 30 min). Cells were washed 3 times in DMEM and incubated with indicated strains using the normal infection protocol with TER monitored for a further 6 h.

### Cell death and detachment assays

The level of infection-induced cell detachment was determined by centrifuging host cells within the supernatant at 500 *g* for 10 min, with two separate PBS washes. This isolated the majority of host cells away from the bacteria, which remained in the supernatant. The cell pellet was lysed with 0.1 M NaOH in PBS and the protein content determined using Bradford reagent (Sigma) assaying at 495 nm. The level of cell necrosis of infected monolayers was determined by incubating with 0.2% (w/v) trypan blue in PBS for 5 min, washing in PBS and visualizing cells using a conventional light microscope. The number of blue cells was recorded per field of view using a ×40 objective lens examining six fields of view per experiment. The level of apoptosis was determined by assaying caspase-9 cleavage using the substrate LEHD-pNA. The liberation of pNA was detected spectrophotometry at 405 nm according to manufacturer's instructions (Calbiochem 218824). As a positive control, cells were incubated in DMEM containing staurosporine (1 µM) to induce apoptosis (Bertrand *et al*., 1994).

### Confocal microscopy

At select times post infection, the TC-7 monolayers were washed gently in PBS and fixed in 2.5% (w/v) paraformaldehyde for 15 min. Cells were then washed and incubated in a solution of phalloidin to stain F-actin as previously described (Dean and Kenny, 2004). Cells were visualized using a Leica SP2 confocal microscope by making a series of optical sections along the z-axis and reconstructing the monolayer as a composite projection. The level of cell detachment was determined using Leica confocal software by calculating the area of cell-free voids as regions of interests in the field of view and expressing as a percentage of the total field.

### Inhibitor assays

All inhibitors were added to the apical and basal wells of polarized TC-7 cells (12–15 days post confluent) for 2 h prior to infection then removed from the apical well (2 washes in DMEM) prior to the addition of bacteria. Bacterial infection and TER monitoring were performed as above. Although the TC-7 cells were washed prior to infection, each inhibitor was assessed for its effect on bacterial growth at the concentration used. No inhibitor used in this study affected bacterial growth in DMEM over the time-course of the experiment (not shown). Working concentrations of inhibitors used in this study and their mode of action is given in Table 1. All inhibitors were purchased from Calbiochem except valinomycin and wortmannin, which were from Sigma.

### Calpain activity

Measurement of calpain activity was performed based on the catalytic cleavage of the synthetic calpain substrate Suc-LLVY-AMC in the presence of Ca^2+^ and the reducing agent TCEP. The liberation of AMC during the reaction was detected at a wavelength of 460 nm. AMC (µM) released was determined using an AMC standard curve. Following infection, TC-7 cells were harvested and washed with PBS, lysed in Cytobuster (Calbiochem) and incubated on ice for 30 min. The lysate was centrifuged at 4°C at 12 000 *g* and the protein content was determined using BCA reagent. Calpain activity was determined using Suc-LLVY-AMC with the calpain activity kit according to the manufacturer's instructions (Calbiochem).

### Statistical analysis

Significance levels within data sets were determined using a one-way anova with a *post hoc* Tukey test indicating significances between individual data points.
